# Inequity in waiting for cataract surgery - an analysis of data from the Swedish National Cataract Register

**DOI:** 10.1186/s12939-016-0302-3

**Published:** 2016-01-19

**Authors:** Goldina Smirthwaite, Mats Lundström, Barbro Wijma, Nina Lykke, Katarina Swahnberg

**Affiliations:** Department of Health and Caring Sciences, Faculty of Health and Life Sciences, Linnaeus University, 391 82 Kalmar, Sweden; Department of Clinical Sciences, Ophthalmology, Faculty of Medicine, Lund University, 221 85 Lund, Sweden; Department of Clinical and Experimental Medicine, Faculty of Health Sciences, Linköping University, 581 83 Linköping, Sweden; Department of Gender Studies, Faculty of Arts & Sciences, Linköping University, 581 83 Linköping, Sweden

**Keywords:** Health inequalities, Social inequity, Cataract extraction, Intersectionality, Gender, Doing gender

## Abstract

**Background:**

Swedish Health and Medical Services act states that good care should be given to the entire population on equal terms. Still studies show that access to care in Sweden differ related to for example gender and socioeconomic variables. One of the areas in Swedish health care that has attracted attention for potential inequity in access is Cataract Extraction (CE). Previous studies of access to CE in Sweden show that female patients have in general poorer vision before they are operated and longer waiting times for CE than male patients. The aim of the study was to describe the waiting times in different patient groups with regards to visual acuity, gender, age, native country, educational level, annual income and whether the patient was retired or still working.

**Methods:**

The study was designed as a register study of 102 532 patients who have had CE performed in Sweden 2010–2011. Linear regression was used to analyse the association between patient characteristics and waiting times. Mean waiting times for women and men were calculated for all groups.

**Results:**

At significance level *p* < 0.05 longer waiting times corresponded to patients having good visual acuity, being of female gender, high age, retired, born outside the Nordic countries and having low income and education. Calculations of mean waiting times for all groups showed that women had longer waiting times than men.

**Conclusions:**

The differences between groups defined, for example, by gender, age, native country, income, education and retirement are statistically significant. We do not consider them as clinically significant, but we consider the consistent pattern that we have found noteworthy in relation to the principle of equity in health care.

## Background

The Swedish Health and Medical Services Act states: “Health and medical services are aimed at assuring the entire population good health and care on equal terms” [1]. Numerous studies, however, indicate that women and men have different access to and experience different quality in health care [2–7]. Studies also report inequalities in the Swedish health care services between different socioeconomic groups and ethnic groups [8], for example, that newer and more expensive drugs are more often prescribed to patients from higher social positions [9–11].

One of the areas in Swedish health care that has attracted attention for potential inequity is Cataract Extraction (CE). Previous studies of access to CE in Sweden show that even though female patients are in the majority, they also have in general poorer vision before they are operated and longer waiting times for CE than male patients [3, 7, 10]. CE is a relatively simple and safe operation, where neither general anaesthesia nor in-patient care is needed. Since cataracts mainly affect elderly people, it can according to The Swedish National Board of Health and Welfare (NBHW) be seen as problematic if old age should be considered as a contraindication for CE. NBHW has pointed out that womens’ longer waiting time and poorer visual acuity when operated could be due to discrimination related to both age and gender [3]. CE, the most common surgical procedure in many European countries [12], is thus an interesting treatment area to study in relation to the principle of equity in health care.

In order to achieve equity in health care, it is important to map different treatments to detect for example inequity in access to care. CE is the most common operation in Sweden, and the fact that large numbers of patients are affected makes it interesting to study. Furthermore, even if earlier studies point to that women in general have longer waiting times than men for CE, it is not known if women’s longer waiting time would persist when adjusted for possible confounders, for example visual acuity, native country, age and educational/income levels. Neither it is known if or how factors as native country, age and educational/income levels affects waiting time for CE.

The aim of the study was to describe the waiting times in different patient groups with regards to visual acuity, gender, age, native country, educational level, annual income and whether the patient was retired or still working.

## Methods

### Description of study population

The material consists of data from 102 532 patients who have had CE performed in Sweden 2010–2011 (Table 1).

**Table 1:** Socio-demographic characteristics for the study population

	Number	Percent	Women	Men
*Native country*				
Sweden	89,563	87.4	53,266	36,297
Nordic countries except Sweden	5726	5.6	3879	1847
European countries except the Nordic ones	4180	4.1	2415	1765
Other countries	3063	2.9	1682	1381
*Education*				
No reported education	1568	1.5	980	588
Elementary school	42,301	41.3	26,169	16,132
Upper secondary school/High school	38,086	37.1	22,463	15,623
University level	20,577	20.1	11,630	8947
*Occupation*				
Retired	84,583	82.5	51,623	32,960
Employed	17,949	17.5	9619	8330
*Age*				
40 – 65	21,024	20.5	11,372	9652
66 – 75	36,552	35.7	22,119	14,433
76 – 85	36,655	35.7	22,666	13,989
*≥86*	8301	8.1	5085	3216
*Annual income in SEK.*				
<70 000	8889	8.7	6217	2672
70*–*149 000	33,190	32.4	28,058	5132
150–249 000	36,296	35.4	17,791	18,505
250–449 000	17,881	17.4	7277	10,604
450*–*649 000	3572	3.5	1056	2516
>650 000	2704	2.6	843	1861
Total	102,532	100	61,242	41,290

*N* = 102 532 (61,242 women and 41,290 men)

Inclusion criteria: Patient >40 years, first-eye operation performed in Sweden during 2010–2011. Exclusion criteria: Second-eye operation, patients ≤40 years, and patients with more than 24 months waiting time.

Patients ≤ 40 years were excluded for the following reasons: Cataracts mainly affect elderly people. For patients younger than 40 years, cataracts are likely to be congenital/juvenile or secondary to other diseases or trauma and in these cases, the normal waiting time rules are not valid. Patients ≤40 years constituted 0.4 % of the patients who had their first CE in 2010–2011.

Patients with more than 24 months waiting time were excluded since such a long waiting time is not normal. The length of the waiting time for those patients in the Swedish National Cataract Register (NCR) is likely to be incorrect due to some error in the registration process or specific agreements, for example, that the patient wants to be operated on by a specific surgeon. Patients with more than 24 months waiting time constituted 0.1 % of the patients who had their first CE in 2010–2011.

### Procedure

#### Data collection

All persons who have a Swedish national registration receive a unique personal identification number from the tax authority. This number is a crucial part of the data collection in this study; it is the nexus in the centre of all data concerning each individual patient, and guarantees that the collected data concerns the right person. This number makes it possible to identify persons in a number of registers.

Data on the patients’ sex, age, visual acuity, which hospital the patient went to, and how long the waiting time (defined below) was for CE for the patient were collected by NCR, which has 98 % coverage of CEs performed in Sweden [13]. The Swedish eye clinics report to NCR voluntarily, and on a regular basis. The data from NCR were sent to Statistics Sweden, who via the patients’ personal identification number could obtain further information on the patient from Statistics Sweden’s Longitudinal Integration Database for Health Insurance and Labour Market studies (LISA). Information obtained from LISA concerned patients’ native country, whether the patients main income source was from occupation or from pension, annual income in Swedish currency (SEK) and educational level.

### Statistical analysis

Linear regression was performed to compare waiting times between the different groups of interest for the study, i.e. sex, age and groups related to class/societal position and native country. A level of 95 % (*p* < 0.05) was regarded as significant. Dummy variables was applied for the several-categorical nominal variables, e g country of birth. All results in the linear regression and also the control variables were mutually adjusted.

The dependent variable was *Waiting time* - defined as waiting time in days from the date of the decision to operate to the date of surgery.

Independent variables defining the groups of interest were:

- *Visual acuity* - used as a quantitative, continuous variable and refers here to visual acuity on the best eye. NCR uses Snellen decimal notation to describe visual acuity. It is a logarithmic scale, which is not suitable for linear regression. Visual acuity was thus converted into LogMAR, which is a linear scale.
- *Gender -* operationalized as a dichotomous variable on a nominal scale. The values were woman or man, and were obtained from the patients registered sex.
- *Age -* operationalized as a quantitative, continuous variable. Age refers here to the patients’ age at the turn of the year before the patient received a medical referral for CE.
- *Native country*- operationalized as a polykotomous variable on a nominal scale, and defined as the country where the patient was born. The variable could have the following values:
  - Sweden,
  - Nordic countries except Sweden,
  - European countries except Sweden and other Nordic countries,
  - Other countries, comprising all countries outside Europe
- *Educational level*- operationalized as a qualitative, polykotomos variable on a ordinal scale and could have the following values for highest reported education:
  - No reported education
  - Elementary school
  - Upper secondary school/High school
  - University level
- *Total annual income*- operationalized as a quantitative, continuous variable. The variable consists of the person’s total registered annual income in Swedish currency, which includes salary, pensions, payment from social insurances and income from capital, interest etc. Total income had a few very high values, (outliers) and to adjust for them a new variable was created. The new variable is the logarithm of total income and has been given the 10 logarithm of its original value.
- *Pension*- operationalized as a qualitative, dichotomous variable on a nominal scale. The values which pensions can have are either that the person is retired or employed/highly active in working life. To create the variable the earned income (mainly salary) has been compared to pension. If the earned income has been higher than the pension, the person has been regarded as employed/working, but if the pension has been higher than the earned income, the person has been regarded as retired.

### Control variables

Two of the independent variables used in the linear regression were not in the scope of the aim for the study, but could have an effect on differences in waiting times between the groups of interest for the study. One of these variables was Month, which refers to which month the patients were placed on waiting list for CE. The earlier month in the study period the patients were placed on waiting list, the longer waiting time they had. This is probably related to a governmental measure called the ‘queue billion’ which was introduced in 2008 and implicates that county councils, which gave patients treatment or operation within 90 days, should have a billion Swedish crowns to share [7].

The other control variable was Hospital, which means which hospital the patient went to. Hospital is operationalised as a qualitative, polykotomos variable on a nominal scale. It is a well known and problematic fact that different hospitals in Sweden have different waiting times for CE. This can be due to for example the number of employed eye-surgeons.

All results in the linear regression have been controlled for “Month” and “Hospital”.”

The result of this analysis is displayed in Table 2. SAS 9.3 was used to perform the linear regressions.

**Table 2:** Extra waiting time to CE in days from date of decision to treat to date of surgery. (Linear regression)

Variable	Extra waiting days	Pr [t]
Gender		
Women compared to men	3. 73	<.0001
Age		
Per year of age	0.40	<.0001
Visual acuity		
Best visual acuity compared to poorest visual acuity^a^	12.07	<.0001
Total annual income		
10-power less income	2.90	<.0001
Education		
No university education	1.28	0.0031
Native country;		
Patients not born in any Nordic country		
Patient born outside Europe	5.59	<.0001
Patient born in Europe	1.92	0.0242
Occupation		
Patient is retired instead of employed.	4.84	<.0001

^a^ Poorest visual acuity: LogMAR 1, best visual acuity LogMAR 0

In order to examine gender differences within the groups defined by visual acuity, age, income, educational level, native country, and whether the patient was working or retired, the mean waiting times were calculated for women and men in each of the groups. Since our material covers the whole population we have not given any *p*-values for these calculations. We have calculated the mean values for the whole populations. When calculating mean values in a full-scale register study, no model is created, and the mean values represent directly the mean values in the population.

For theoretical reasons we have given *p*-values concerning the linear regression, since the linear regression creates a model and the *p*-value indicates the likelihood that the model corresponds to the population.

### Ethics

The authors have considered the ethical aspects of the study and followed the guidelines of the Helsinki Declaration. The study is approved by the Regional Ethic Board in Linköping (Dnr 2010/380-31) and the Swedish Central Ethical Review Board (Dnr O 5–2011).

## Results

A total of 61 242 women and 41 290 men were included, and the socio-demographic characteristics are presented in Table 1.

There were notable differences between female and male patients regarding income. Although men were in a minority in the material, they were in an absolute majority in each one of the four highest income groups as presented in Table 1.

At the same time, the group of patients with a total annual income under 150 000 SEK consisted of 82.5 women and 17.5 % men. The median income (not shown in table) was 134 400 SEK for women and 207 900 SEK for men.

Concerning the results of the linear regression analyses, they show that three of the variables - *educational level*, *native country* and *hospital -* contained some non-significant categories.

For *educational level,* no significant difference was found between the following categories: No reported education, Elementary school and Upper secondary school/High school.

The only category which differed significantly from the other categories in this variable was University level.

For *native country* the difference between patients born in Sweden and patients born in other Nordic countries was not significant, and for *hospital,* there were two hospitals that had no significant difference in waiting times in relation to each other.

According to the linear regression, following patients had statistically significant longer waiting time than others, all other variables the same: patients with good visual acuity, patients of female sex, patients in higher age, patients with lower income, patients not having education at university level and patients who were retired. Patients born outside the Nordic countries (in Europe and in countries outside Europe) had longer waiting times than patients born in the Nordic countries, including Sweden. Between Sweden and the other Nordic countries, there were no significant differences in waiting time. The longer waiting times related to these factors is presented in Table 2 in the column Extra waiting days. Extra waiting days is defined as the days a patient from one group, for example women, wait longer than a patient from another group, in this case men, if all the other variables are the same. Longer waiting times were also related to which month the patient was placed on waiting list and which hospital the patient went to All results in the linear regression have been controlled for these two variables, but these two variables are not shown in Table 2. Since we controlled for these two variables we can conclude that waiting time differences related to for example gender, native country and income not are confounded by Month or Hospital but persist even when adjusted for these variables.

**Fig. 1:**
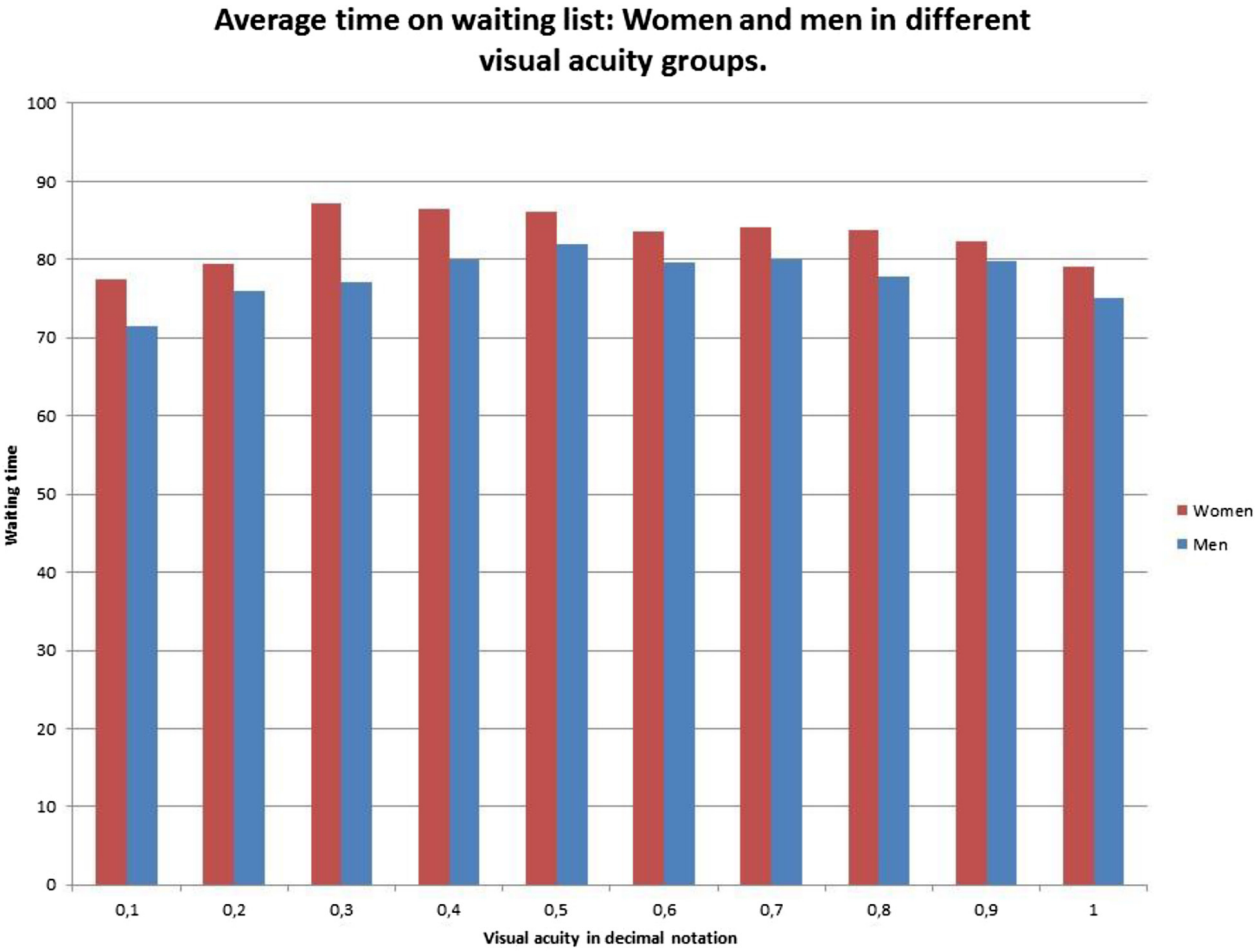
Average time on waiting list: Women and men in different visual acuity groups

Waiting time differences were found not only *between* the groups shown in Table 2, but also between female and male patients *within* all of these groups. For example, as shown in Fig. 1, at all levels of visual acuity women had somewhat longer waiting times than men. All the diagrams show average waiting time for women and men, in crude numbers.

**Fig. 2:**
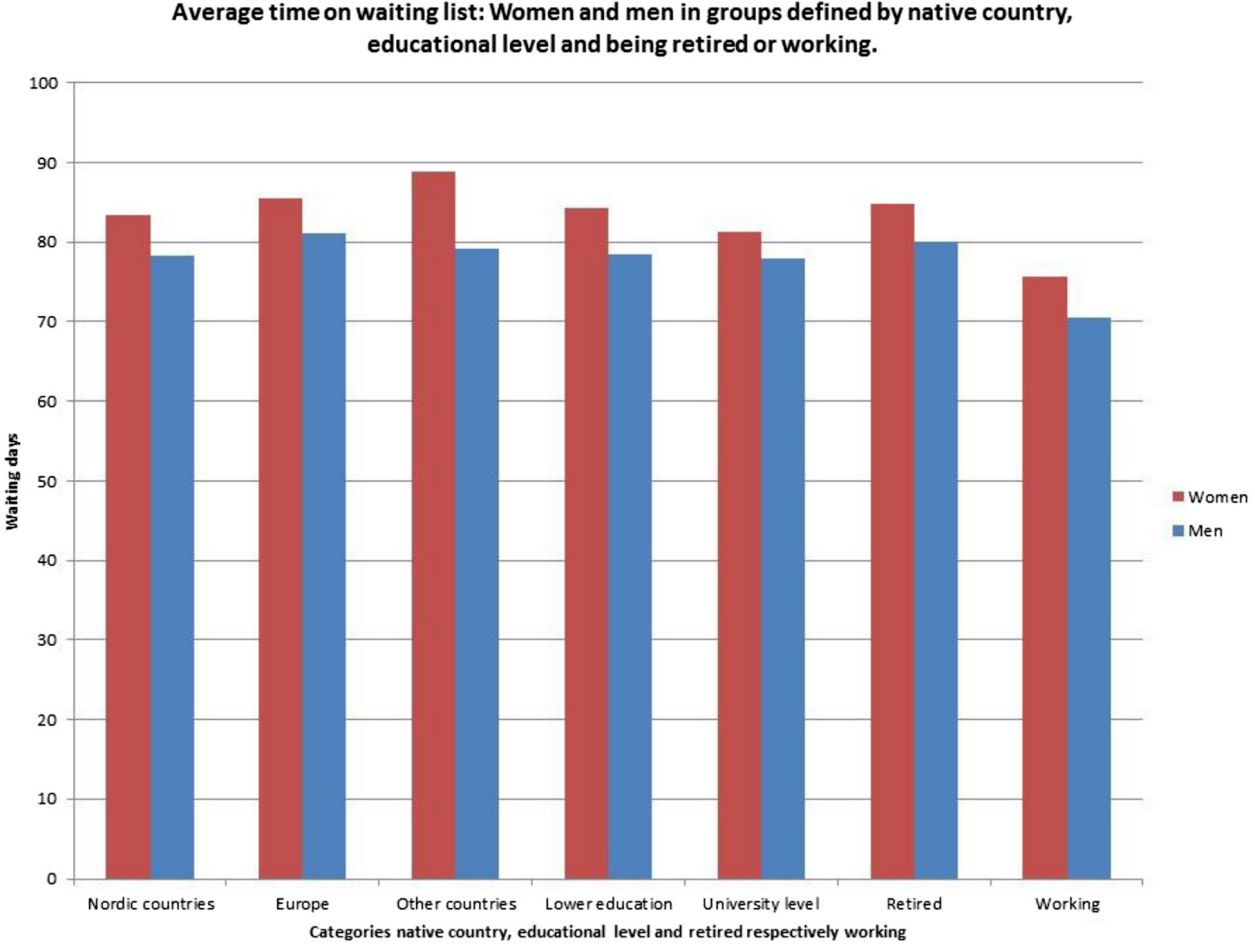
Average time on waiting list: Women and men in groups defined by native country, educational level and being retired or working

The result was similar concerning groups related to patients’ native country, educational level and whether patients were retired or not. In all these groups, as shown in Fig. 2, women had longer waiting times than men.

**Fig. 3:**
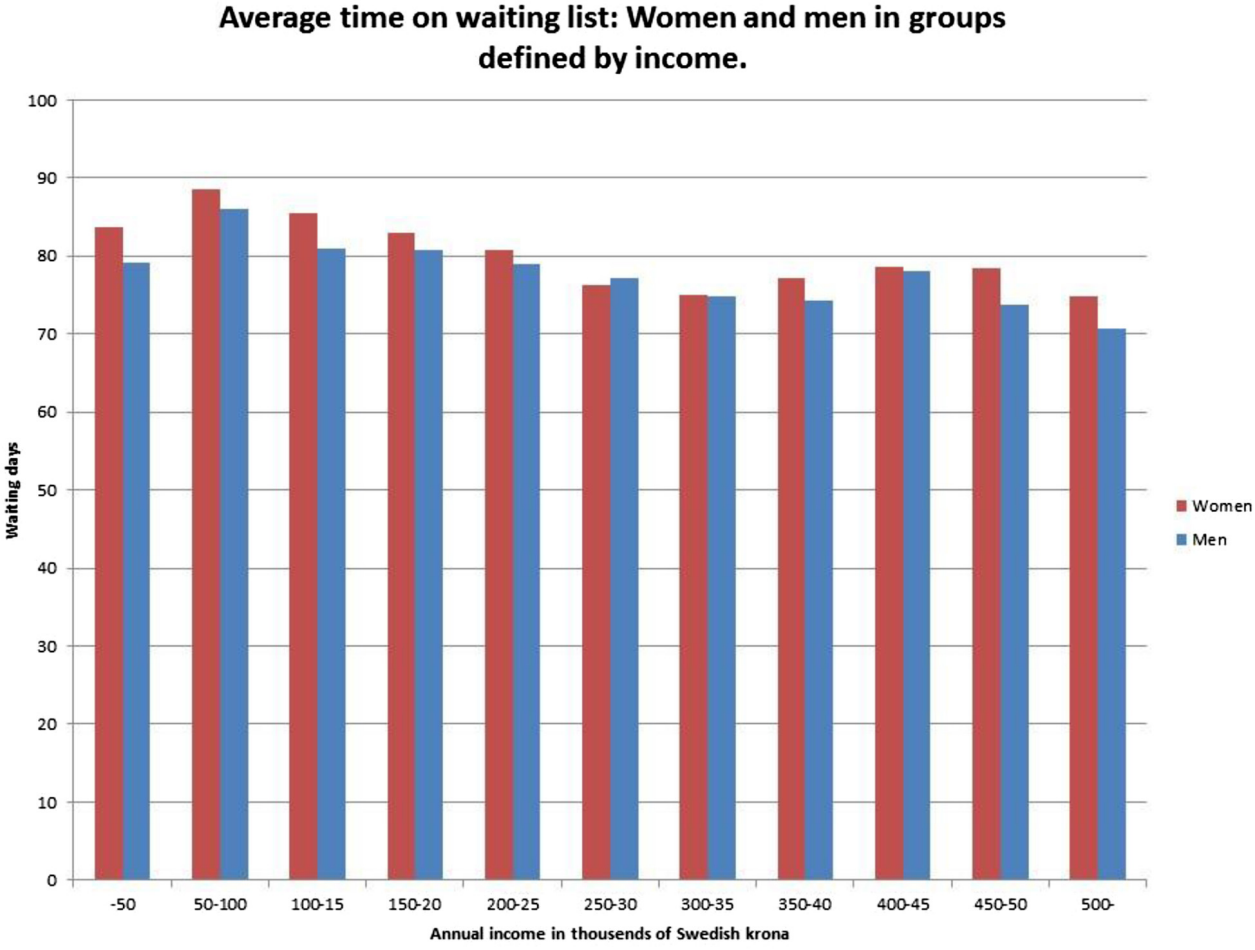
Average time on waiting list Women and men in groups defined by income

**Fig. 4:**
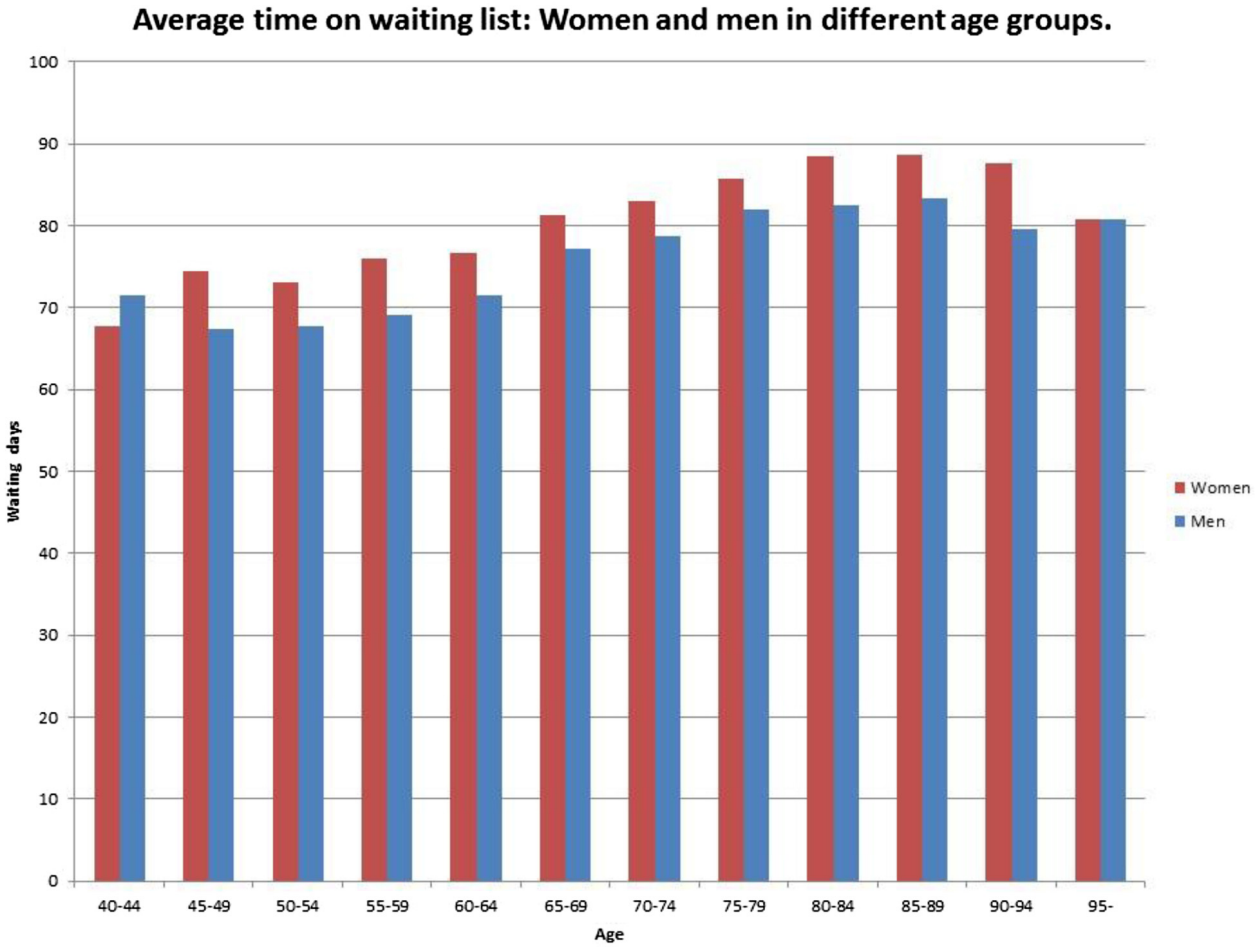
Average time on waiting list: Women and men in different age groups

Women had also longer waiting times than men where the great majority of income groups (Fig. 3) and age groups (Fig. 4) are concerned.

## Discussion

Gender-related differences in access to CE in Sweden have decreased over the last decade, which may well be related to governmental measurements, for example, a waiting time guarantee [7]. Even if waiting times for CE in general have been reduced, there still remain waiting time differences related to, for example, gender. These differences are on average relatively small. Even if our results are *statistically* significant, we do not consider them to be *clinically* significant, but we think that our study points to more general tendencies in Swedish health care, which can have clinical significance in other areas. Our results indicate, similarly to those found in other studies, a lack of equity in Swedish health care, for example, that women, ethnic minorities and persons with low income have poorer access to several kinds of health care [8, 9, 11, 14–18].

The differences in waiting times, which our study show, could theoretically be related to intersectionality [19, 20]. Intersectionality is a theoretical and methodological tool for analyzing how specific kinds of power differentials interact and produce societal inequities [21]. Intersectional researchers often points to the importance of not just adding subordinated categories to each other in order to grasp the situation of persons living in intersections of different marginalised or disadvantaged categories [21–24]. Instead the categories are seen as mutually transforming and shaping each other. However, we find our study to be in line with what McCall has defined as the intercategorical approach within intersectional theory, were scholars provisionally adopt existing analytical categories in order to document, for example, relationships of inequity among social groups [20].

According to our linear regression model, a female patient who is born outside Europe and who does not have education at university level would have 10.6 days longer waiting time than a male patient who is born in a Nordic country and is educated at university level - if all other variables are the same. The waiting time difference could be further increased (or decreased) if differences in, for example, income and age are added. By adding together waiting times for different groups we show the consequences in waiting time, for example, for patients who at the same time belong to several groups which each have prolonged waiting times. Even if the scale is additive, our research does not entail an additive assumption when it comes to assessing how intersecting inequalities may affect other parts of the patients’ lives.

We can see, for example, in the diagrams how being a woman intersects with being born outside Europe, and that patients in this specific intersection between gender and native country have longer waiting times than for example women born in the Nordic countries. We can also see that women born in Nordic countries have longer waiting times not only compared with men in Nordic countries, but also with men born in other European countries and in countries outside Europe.

The differences in waiting times could theoretically also be related to a doing gender perspective [25–27]. According to a doing gender perspective, gender is perceived as something people do in their everyday social interactions when they act in relation to norms and notions of gender. Research shows that conceptions about gender can influence how medical data are interpreted differently according to the patient’s gender, and in ways which are not necessarily motivated medically. Research also shows that men and women may get different access to treatment through ways which are not motivated by medical facts [2–5, 8–11, 14–18]. The doing gender processes includes both staff and patients, and the gender differences in waiting times presented in this study could be interpreted as consequences of processes of doing gender. We cannot, however, on the basis of this quantitative study analyse in detail how these processes may influence gender differences in waiting times, we can only state that such differences are not coincidental.

In the linear regression we have measured costs in terms of extra waiting times related to different groups. The only costs we find motivated by medical and ethical reasons concerns good visual acuity. It is in accordance with the Swedish principles of equity in health care to prioritize patients with greater needs [28]; in this case those with poorer visual acuity had shorter waiting times. Since we have adjusted for visual acuity, we can exclude that differences in visual acuity is a confounder, for example, in terms of gender-related or age-related differences in waiting time. It is also interesting to note, as shown in Fig. 1, that for each and every step in the Snellen decimal notation scale for visual acuity, women have longer waiting times than men. The gender differences in waiting time can thus not be explained by gender differences in visual acuity. A strength in our study is that we have adjusted for both visual acuity and other factors that in statistical analyses have proved to be relevant for waiting time (i.e. which month the patient was placed on waiting list for CE, and which hospital the patient went to).

We consider that the pattern found in our results is notable in relation to the principle of equity in health care. We cannot find any medical reasons why women should have a longer waiting time than men, why patients born outside the Nordic countries should wait longer than others, why poorer patients and patients without education at university level should wait longer than richer or higher educated patients. And last, but not at least, why patients living in intersection between categories with longer waiting times – for example being a woman and being born outside Europe - should have poorer access to CE than other patients.

## Conclusions

Linear regression analyses show that there are differences in waiting time for CE in Sweden between different groups. Longer waiting times corresponded to patients having good visual acuity, being of female gender, high age, retired, born outside the Nordic countries and having low income and education. Calculations of average waiting times for men and women show that women had longer waiting times than men in all groups of visual acuity and native countries and in the great majority of age groups and income groups. Women also had longer waiting time than men both in the group of retired patients, and in the group of patients who were still working. Women also had longer waiting time than men both in the group with education at university level, and in the group without such education. The pattern found in our results is notable in relation to the principle of equity in health care.

## Abbreviations

CE: cataract extraction
NBHW: Swedish National Board of Health and Welfare
NCR: Swedish National Cataract Register
