# Erratum to: Inequity in waiting for cataract surgery - an analysis of data from the Swedish National Cataract Register

**DOI:** 10.1186/s12939-016-0355-3

**Published:** 2016-04-11

**Authors:** Goldina Smirthwaite, Mats Lundström, Barbro Wijma, Nina Lykke, Katarina Swahnberg

**Affiliations:** Department of Health and Caring Sciences, Faculty of Health and Life Sciences, Linnaeus University, Kalmar, 391 82 Sweden; Department of Clinical Sciences, Ophthalmology, Faculty of Medicine, Lund University, Lund, Sweden; Department of Clinical and Experimental Medicine, Faculty of Health Sciences, Linköping University, Linköping, 581 83 Sweden; Department of Gender Studies, Faculty of Arts & Sciences, Linköping University, Linköping, 581 83 Sweden

Unfortunately, after publication of this article [1], it was noticed that Figure 3 (Fig. 1 here) contained an error. The axis labels for the x axis allowed overlapping of the groups of data. The corrected figure can be seen below and the original article has been updated to reflect this change.

**Fig. 1:**
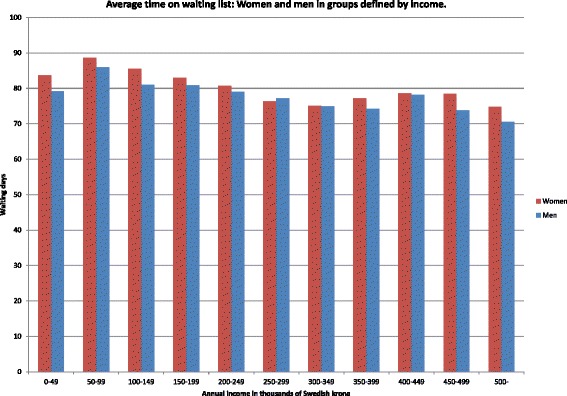
Average time on waiting list Women and men in groups defined by income

## References

[1] Smirthwaite G, Lundström M, Wijma B, Lykke N, Swahnberg K (2016). Inequity in waiting for cataract surgery - an analysis of data from the Swedish National Cataract Register. Int J Equity Health.

